# Contextual effects: how to, and how not to, quantify them

**DOI:** 10.1186/s12874-024-02152-2

**Published:** 2024-02-13

**Authors:** Tobias Saueressig, Hugo Pedder, Patrick J Owen, Daniel L Belavy

**Affiliations:** 1 Department of Applied Health Sciences, Division of Physiotherapy, Hochschule für Gesundheit (University of Applied Sciences), Gesundheitscampus 6-8, 44801 Bochum, Germany; 2 Physio Meets Science GmbH, Johannes Reidel Str. 19, 69181 Leimen, Germany; 3 Population Health Sciences, Bristol Medical School, University of Bristol, Canynge Hall 39, Whatley Road, Bristol, BS8 2PN UK; 4 Eastern Health Clinical School, Monash University, Melbourne, Victoria Australia

**Keywords:** Methodology, Meta-analysis, Contextual effects, Placebo effects

## Abstract

The importance of contextual effects and their roles in clinical care controversial. A Cochrane review published in 2010 concluded that placebo interventions lack important clinical effects overall, but that placebo interventions can influence patient-reported outcomes such as pain and nausea. However, systematic reviews published after 2010 estimated greater contextual effects than the Cochrane review, which stems from the inappropriate methods employed to quantify contextual effects. The effects of medical interventions (i.e., the total treatment effect) can be divided into three components: specific, contextual, and non-specific. We propose that the most effective method for quantifying the magnitude of contextual effects is to calculate the difference in outcome measures between a group treated with placebo and a non-treated control group. Here, we show that other methods, such as solely using the placebo control arm or calculation of a ‘proportional contextual effect,’ are limited and should not be applied. The aim of this study is to provide clear guidance on best practices for estimating contextual effects in clinical research.

**Supplementary Information:**

The online version contains supplementary material available at 10.1186/s12874-024-02152-2.

## Introduction

The importance of contextual effects in clinical care remains contentious [1, 2]. We define ‘contextual effect’ as the influence of contextual elements on the clinical outcome (see the ‘Definition’ section for more details). A Cochrane review published in 2010 concluded that placebo interventions do not achieve important clinical effects overall, but that placebo interventions can influence patient-reported outcomes such as pain and nausea [3]. When pain was assessed using a binary “yes-no” outcome, there was no discernible placebo effect (risk ratio: 0.92, 95% confidence interval (CI): 0.77,1.11) based on data from six trials involving 1207 participants. However, when pain was evaluated on a continuous scale, the authors found a modest effect (standardized mean difference (SMD): -0.28, 95% CI: -0.36, -0.19) from data gathered across 60 trials with 4154 participants [3]. In practical terms, this small effect corresponds to an approximately five-unit change on a pain scale ranging from zero to 100. This conversion was achieved by re-expressing the SMD of -0.28, onto a 0-100 pain scale with an assumed standard deviation of 20 points [1, 4].Although this effect is statistically significant and may be meaningful across multiple patients, the size of the effect is such that it is unlikely to be noticeable by the average individual patient [1].

Notably, when relying on patient-reported outcomes, it is increasingly difficult to discriminate between the patient-reported effects of placebo and response bias [3]. Response bias is the tendency of people to answer questions in a way that is not accurate or truthful for some reason(s) (e.g., social desirability bias, acquiescence bias, demand characteristics, fear of judgement or stigma, recall bias, cultural bias, cognitive bias) [5]. Compared to a previous Cochrane review [3], more recent systematic reviews [6–17] estimated greater proportional contextual effects for pain that ranged from 50 to 75% of the total treatment effect. Naturally, this raises the question of how this discrepancy can be explained.

We contend that these differences in estimates arise from inappropriate meta-analytical methods employed to quantify contextual effects. The objective of this study is to elucidate contextual effects and offer best practices for robust estimation in comparison to the overall treatment effect. Furthermore, we provide insights into why certain methodologies may not be suitable for assessing the contextual effects. Discerning contextual effects in clinical practice is important to gain an understanding of their magnitude and causal pathways.

## Definitions

**Fig. 1:**
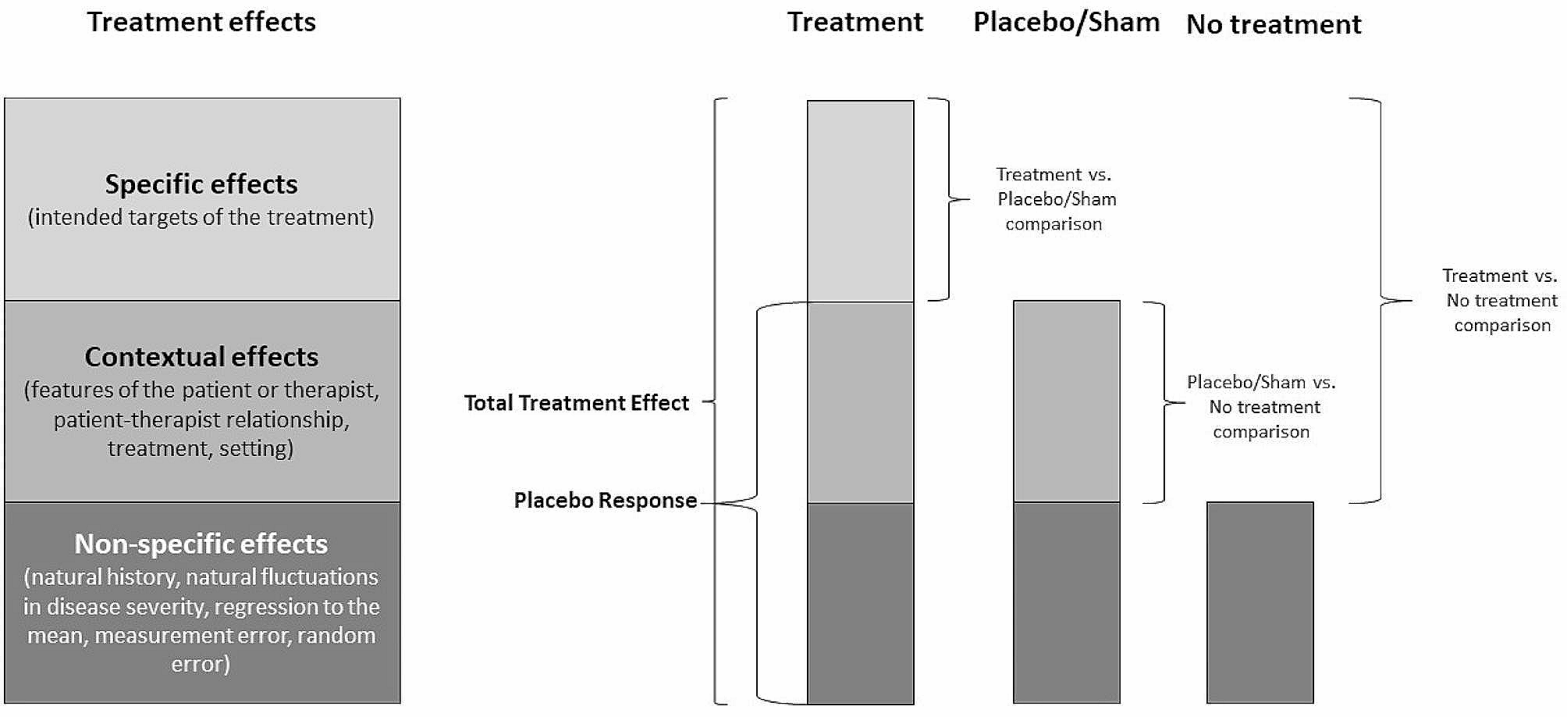
Total treatment effect encompasses the specific effects of treatment, contextual effects, and nonspecific effects. The addition of contextual and non-specific effects is called the placebo response. The treatment vs. placebo/sham comparison controls for contextual and nonspecific effects to isolate the specific treatment effect. The placebo vs. no-treatment comparison controls for nonspecific effects to isolate contextual effects. The treatment vs. no-treatment comparison controls for non-specific effects to isolate contextual and specific treatment effects. Adapted from the study by Cashin et al. [18]

The effects of medical interventions (i.e., total treatment effect) are commonly divided into three components: specific, contextual, and non-specific (Fig. 1) [3, 18, 19].

### Specific effect

The specific effect stems from the treatment itself and arises from the physiological mechanism of action (e.g., the action of a drug on a specific receptor in the body). It is calculated by subtracting the contextual and non-specific effects from the total treatment effect [19].

### Contextual effect

Contextual effects are changes in the clinical outcome that result from exposure to factors related to the context of the healthcare setting. These factors include patient-related aspects (e.g., treatment expectations), therapist-related factors (e.g., friendliness) [20], patient-therapist relationships [21], and intervention settings [22]. Contextual effects produce a treatment effect independent of the specific effects of the intervention and are synonymous with “placebo-related effects,” occurring even in the absence of the inert treatment [23]. For an in-depth exploration of the mechanisms underlying the contextual effects, refer to Enck et al. [24].

### Non-specific effect

Non-specific effects are associated with the natural course of the disease, including natural fluctuations in disease severity [25], regression to the mean [26], measurement error [27], random error [27], spontaneous remission [28–33], and the Hawthorne effect [34]. Unlike specific effects, nonspecific effects are not inherent to treatment and occur naturally over time.

### Placebo

A placebo is an intervention that is presumed to lack a specific effect, i.e., an effect for which there is an empirically supported theory of its mechanism of action, on the condition of interest, but that has been shown to be superior to no intervention [35].

### Placebo response

The placebo response is defined as “[…] *health changes that result after the administration of an inactive treatment (i.e., differences in symptoms before and after treatment), encompassing natural history and regression to the mean*” [36]. Therefore, placebo response refers to contextual and non-specific effects. Some authors equate the placebo response with contextual effects [7], which is misleading given that contextual effects are generated through exposure to contextual factors (e.g., expectations and setting of the intervention) alone. Therefore, non-specific effects should be differentiated from contextual effects, as the former occur irrespective of the treatment provided. Contextual effects are intricately tied to the specific treatment provided, meaning that they are influenced by the unique characteristics and components of the intervention [37].

### Proper estimation of contextual effects: comparing a no-treatment or a ‘placebo-control group’ with a placebo group

**Fig. 2:**
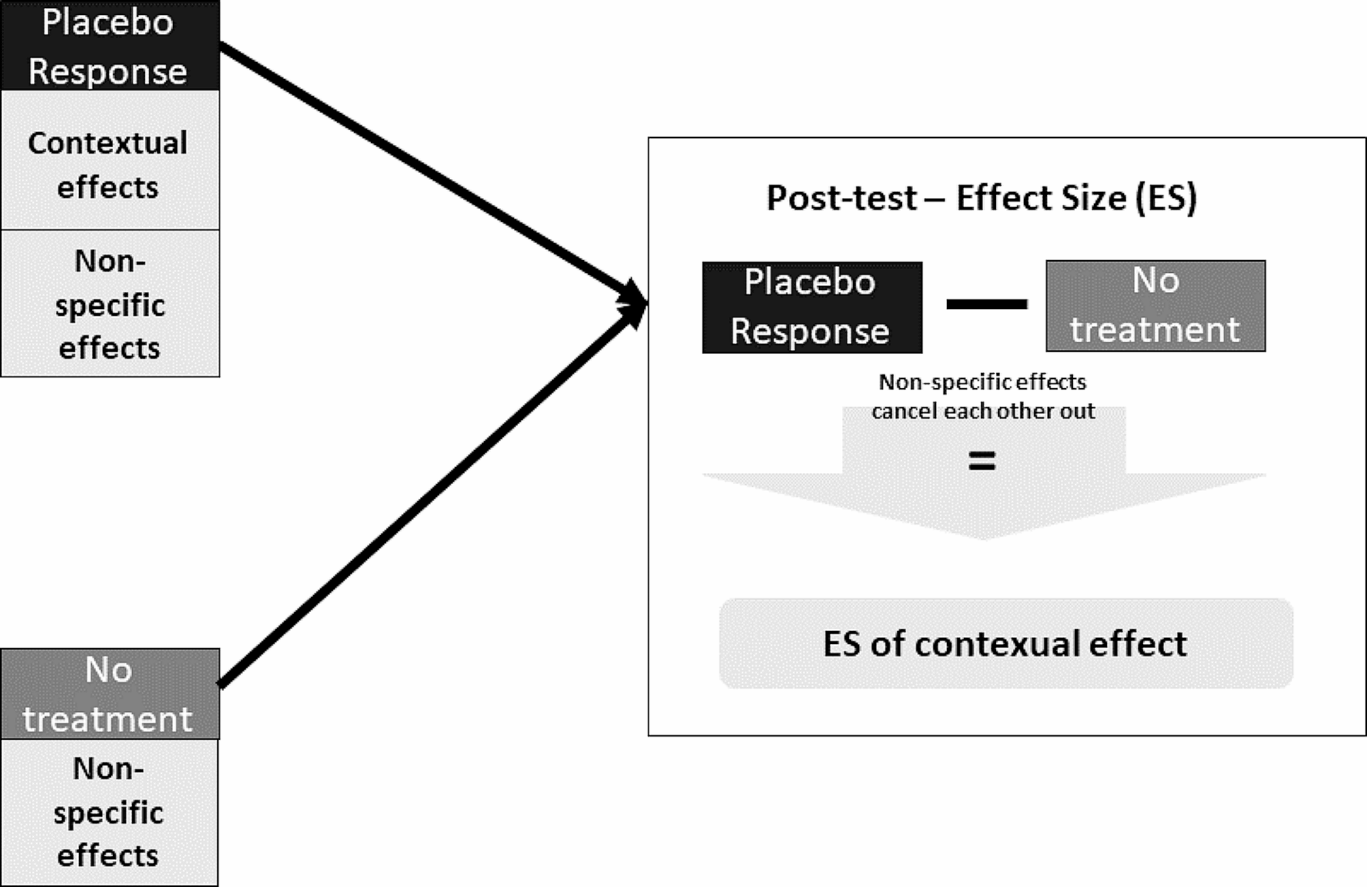
Proper estimation of contextual effects by calculating the difference between the placebo and no-treatment control groups. Non-specific effects cancel each other out and the effect size shows the magnitude of the contextual effect

In a seminal paper, Gøtzsche [35] defined the contextual effect as “*the difference in outcome between a placebo treated group and an untreated control group in an unbiased experiment*.” Notably, this definition is based on groups rather than individuals, as it is often not possible to observe the counterfactual at the individual level (i.e., results of the untreated individual). A randomized crossover design would be an exception in this case, as the individual is the unit of analysis. Gøtzsche stated that an untreated control group was required to adjust for non-specific effects when measuring contextual effects. It is assumed that by subtracting the results of the placebo group from the untreated control group, non-specific effects are negated; therefore, only the contextual effects associated with the placebo group remain (see Fig. 2). Gerdesmeyer et al. [38] contend that this design leads to biased estimates, as an untreated control group may increase the risk of bias (e.g. attrition bias, response bias, compensatory rivalry, resentful demoralization) when assessing outcome measures [38]. One way to mitigate this problem is to use a (modified) Zelen Design [24, 39, 40], which is a modification of the three-arm trial design described above. This design separates the recruitment of patients for an observational study from the recruitment of patients for an interventional trial and allows monitoring of the natural course of the disease without randomizing participants to a no-treatment control group [24]. We would like to emphasize that while the (modified) Zelen Design mitigates some of the inherent biases (e.g., attrition bias, response bias, compensatory rivalry, and resentful demoralization) associated with employing a three-arm trial design, it does not completely eliminate them.

Alternatively, examining two placebo-controlled groups may allow for quantification of contextual effects. In this example, participants in one group are told that they are receiving the “real” treatment, whereas the other group is told that they are receiving a placebo treatment that has no effect [38]. Gerdesmeyer et al. [38] contend that this is a better design to study the actual placebo effect, as an untreated control group may increase the risk of bias (e.g. attrition bias, response bias, compensatory rivalry, resentful demoralization) when measuring outcome measures [38]. We believe that the risk of these biases also applies to the randomized controlled trial design proposed by Gerdesmeyer et al. [38], as patients who were told that they would be treated with an actual placebo treatment that has no effect might also develop compensatory rivalry (i.e., participants in the group not receiving the experimental treatment feel disadvantaged, disappointed, or left out, and therefore seek similar or alternative treatments on their own) [41] and/or resentful demoralization (i.e., participants in the control group became resentful of not receiving the experimental treatment) [42]. Collectively, these factors may increase the risk of attrition bias, with a corresponding drop out in the placebo group, which was told the truth about their treatment. In this example, participants in one group are told they are receiving the “real” treatment (placebo active group), whereas the other group are told they are receiving a placebo treatment that has no effect (placebo control group) [34]. Another limitation of this design is that it does not allow the exclusion of non-specific effects. Although these effects cancel each other out, the contextual effect is not entirely isolated.

An alternative design that provides a thorough evaluation of contextual effects is a three-arm study as described in the literature [43, 44]. This design includes two placebo groups, as proposed by Gerdesmeyer and colleagues [38]: one placebo active group where participants believed they were receiving the actual treatment, and a placebo control group where participants were informed that they were receiving a placebo with no therapeutic effect. Comparing these groups allows for consideration of any remaining physiological effects related to the sham treatment, such as the calming impact of a non-active cream. Additionally, a third group that received no treatment (the natural history group) was included to control for non-specific effects. This tripartite approach enables a detailed analysis of contextual effects, separating the placebo effect from other confounding factors linked to sham treatment, while also considering non-specific effects. However, this design is not immune to the biases present in other study designs. An overview of the study designs is presented in Table 1.

**Table 1:** Key recommendations for studies attempting to assess contextual effects

Possible study design	What does it do?	Potential limitations
Three arm study: (1) intervention (2) placebo/sham (3) untreated control.	• Adjusts for non-specific effects, via untreated control, when measuring contextual effects.	• Untreated control group may increase the risk of bias (e.g. attrition bias, response bias, compensatory rivalry, resentful demoralization) when measuring outcomes. • Assumes that an additive model of treatment effects is true or at least that this assumption does not impact the statistical analysis.
(Modified) Zelen Design – three arm study, randomization before informed consent: (1) intervention (2) placebo/sham (3) untreated control.	• Same as three arm design and lessens the risk of bias through attrition bias, response bias, compensatory rivalry and resentful demoralization. • Allows monitoring of natural disease course without randomizing participants to a no-treatment control group.	• Fewer research questions are ethically appropriate for this design • There still might be risk of bias (e.g. attrition bias, response bias, compensatory rivalry, resentful demoralization) when measuring outcomes. • Assumes that an additive model of treatment effects is true or at least that this assumption does not impact the statistical analysis.
Two arm study: (1) placebo/sham: participants informed that they are receiving a “real” treatment (2) placebo-control group: informed they are receiving a placebo treatment that has no effect.	• The placebo-control group is used to control for potential confounders related to the treatment context. • Might lessen risk of bias as both groups receive “treatment”.	• As patients are told that they are treated with an actual placebo treatment that has no effect they might also develop compensatory rivalry and/or resentful demoralization. • Assumes that an additive model of treatment effects is true or at least that this assumption does not impact the statistical analysis.
Three arm study (1) placebo/sham: see above (2) placebo-control group: see above (3) untreated control.	• In comparison to two arm study (row above), inclusion of an untreated control group to control for non-specific effects. • Might lessen risk of bias as both groups receive “treatment”.	• As patients are told that they are treated with an actual placebo treatment that has no effect they might also develop compensatory rivalry and/or resentful demoralization. • Untreated control group may increase the risk of bias (e.g. attrition bias, response bias, compensatory rivalry, resentful demoralization) when measuring outcome measures. • Assumes that an additive model of treatment effects is true or at least that this assumption does not impact the statistical analysis.

Based on the model presented thus far, we assume that the effects of non-specific effects, placebo effect, and treatment effect are additive, indicating that they do not depend on or interact with each other [45]. Some authors [46–49] challenged the idea that the effects of (treatment) are additive. They argued that the placebo effect and non-specific effects can influence each other. This means that placebo can either enhance or reduce the effects of other factors, such as the natural healing of the body [45]. Following Senn [50], we recommend using a simpler model (i.e., additive model) unless (1) real evidence indicates that this assumption is untrue and (2) if continuing to naively assume that this assumption is true, it will cause a problem in the statistical analysis.

Overall, we contend that a no-treatment group or ‘placebo-control group’ (i.e., an unblinded group that is aware that they are receiving a placebo/sham intervention) should be used to measure contextual effects; however, there is a need to consider the potential risk of bias associated with this experimental design. For an overview of the different study designs used to measure contextual effects, please refer to Table 1.

### Inappropriate method: Four meta-analyses evaluated only the placebo arm

We identified four meta-analyses that inappropriately evaluated only the placebo arm [10, 11, 15, 16]. Measuring the within-group changes of the placebo arm between baseline and follow-up does not measure contextual effects because the measurement contains non-specific effects (e.g. statistical factors, biological properties of the disease, and psychological aspects of receiving attention by clinical staff) [51]. Some researchers further dichotomize their continuous results using an arbitrary response threshold to identify responders and non-responders to placebo intervention [10, 11], which is considered problematic by some statisticians, as it leads to the use of arbitrary thresholds that influence effect estimates [52, 53]. Nevertheless, responder analysis is considered by some researchers as a valid method of analysis, especially in the realm of pain research [54, 55]. We also contend that it is difficult to identify a clear biological/clinical rationale for dichotomous response versus non-response in the placebo group.

We reject the concept of considering the within-group changes in the placebo group as a placebo response. Specifically, there is no need to quantify the placebo response, as it has limited relevance regarding the magnitude of contextual effects. Rather, quantifying this response creates a potential for misunderstanding, whereby research consumers may mistakenly believe that contextual effects are a large component of the total treatment effect, and subsequently deviate from engagement with evidence-based treatments with established effectiveness.

### Inappropriate method: attempting to quantify proportional contextual effect used in eight meta-analyses

Eight meta-analyses [6–9, 12–14, 17] attempted to quantify contextual effects via the proportional contextual effect (PCE). This method of measuring contextual effects was first proposed by Zhang et al. [9, 56] and was derived by comparing an active treatment group with a placebo control group. The total treatment effect was measured as an effect size by the active treatment group, and the contextual effect was measured as an effect size by the placebo group. As an effect size, the mean change from baseline in standard deviation (SD) units was calculated for each group. The PCE is then calculated by dividing the effect size of the placebo group by the effect size of the active treatment group [PCE = $(\frac{Improvement\,of\,the\,outcome\,in\,the\,placeo\,group}{Improvement\,of\,the\,outcome\,in\,active\,group}= \frac{{d}_{placebo}}{{d}_{active}})$]. The standard error (SE) SE corresponding confidence interval were calculated according to the effect size of the response ratio [57]. The authors [9] stated that theoretically, the PCE should range from 0 (0% contribution of contextual effects) to 1 (100% contribution from contextual effects); however, the effect size occasionally exceeds 1, which is interpreted as a 100% contribution from contextual effects. Notably, this method excludes trials in which patients in either the treatment or placebo group worsened from the baseline from analysis [9], which subsequently introduces an inherent risk of bias. The PCE is log-transformed for each study, and the SE are calculated according to Hedges et al. [57]. The log-transformed PCE and log SE is then pooled via meta-analysis, and the summary effect is then back-transformed via exponentiation (for example, see Supplement 1).

To explain why we contend that PCE does not measure the proportion of the total treatment effect attributed to contextual effects, an understanding of the estimation of the treatment effects is warranted. Treatment effects are most often statistically modeled on an additive scale [58, 59]. This means that an improvement on a quality-of-life scale (0-100 points) from 50 to 70 points is an additional 20 points and represents constant improvement. By contrast, a multiplicative or proportional treatment effects model may conclude that the quality-of-life scale improves by 15%. Percentage change is inherently limited by the reliance on baseline values whereby a change from 50 to 57.5 points or 70 to 80.5 points both represent a 15% improvement, despite differences in raw changes of 7.5 vs. 10.5 points. Notably, multiplicative modeling is often used when the underlying data require log-transformation or when there are other plausible biological reasons to use a multiplicative model (e.g., a quality of life scale that is composed of various domains that are summed together; if the treatment has an effect on multiple scales, then the overall effect will be multiplicative) [58]. PCE uses a multiplicative model based on the response ratio, which is also known as the ratio of means [57, 60, 61]. Here, we show that PCE only calculates a treatment effect (as a percentage/proportion) rather than the contextual effect relative to the total treatment effect.

**Fig. 3:**
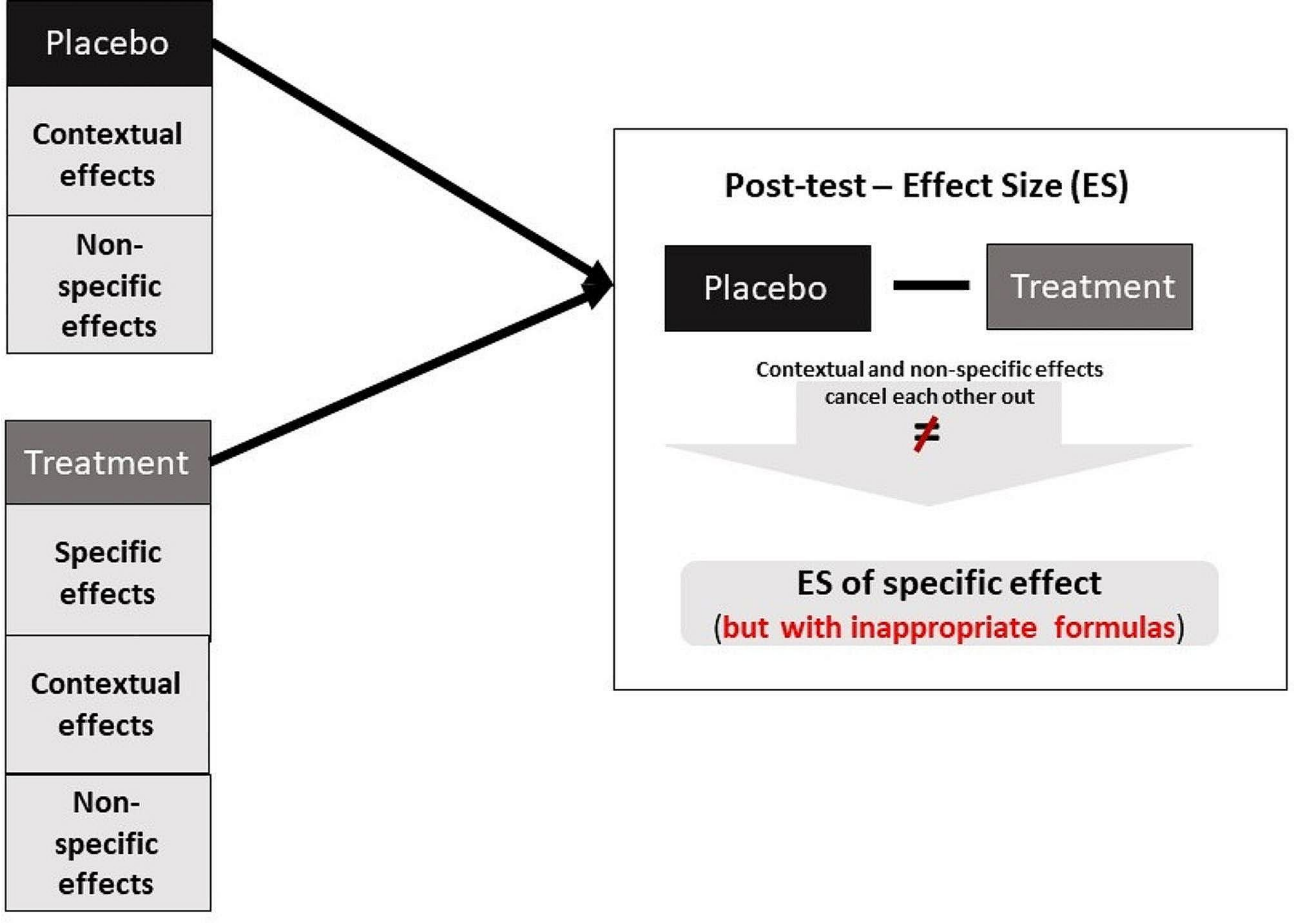
Inappropriate method for measuring contextual effects using the proportional contextual effect method. In this case, a specific treatment effect (on a multiplicative scale) is calculated and not a “proportional contextual effect” because the comparison of treatment versus placebo group controls for non-specific and contextual effects. It should be noted that inappropriate formulas for the calculation of the treatment effect and its variance were used

The PCE is equal to $\frac{{d}_{placebo}}{{d}_{active}}$. This is then log-transformed for pooling purposes. This turns, according to the laws of logarithms (see Supplement 2), a division into subtraction: ln(PCE) = ln($\frac{{d}_{placebo}}{{d}_{active}}$) = ln(${d}_{placebo}$) – ln(${d}_{active}$). One can clearly see that one calculates a treatment effect between the placebo group and the active treatment group (see also Fig. 3). When calculating the treatment effect between a placebo group and a treatment group, the placebo group is used to precisely control for non-specific and contextual effects of the treatment group to obtain an unbiased estimate of the treatment effect [62]. The results obtained are specific treatment effects expressed on a multiplicative scale and **not** a proportion of the contextual effect of the intervention. A further problem with these calculations is that the response ratio is not suitable for change from baseline measures because they can be negative (i.e., the logarithm of a negative number is not defined) [4]. This is why negative changes from baseline are excluded from the calculation of the PCE, which increases the risk of bias due to study exclusion and overall reduces statistical power. Notably, it is possible to calculate a response ratio/ratio of means with change from baseline scores; however, this entails different calculations using the ratio of the ratio of means with appropriate standard errors [58]. One can also question standardization by the SD units of the mean changes because the response ratio/ratio of means is unitless [60] and is confounded by the method of standardization and the accuracy of the method used to estimate the (standardising) SD. Any standardization is superfluous in this case. PCE is also not (naturally) normed to a percentage value, which makes it nonsensical to interpret it as such. To see this, we can make the following example: we have two groups: one active group with a (standardized) mean change of ${d}_{active}$ (4 − 1.5)/1 = 2.5 and a placebo group with a mean change of ${d}_{placebo}$ (4.1 − 3)/1.1 = 1. The PCE is exp[(ln(2.5/1)] = 2.50, which implies that 150% of the total treatment effect is explained by the contextual effect. This shows that the measure is limited in that it does not measure what it purports to measure. In summary, PCE does not measure contextual effects, and we do not recommend that it be employed in future research that attempts to quantify contextual effects.

## Conclusion

The difference between a placebo group and a no-treatment group or an ‘placebo-control group’ can be used to measure contextual effects; however, the inherent biases associated with these designs should be considered. Using the placebo arm alone or calculating PCE represent inferior and therefore inappropriate methods for quantifying the contextual effect and should be retired from use in future studies.

### Electronic supplementary material

Below is the link to the electronic supplementary material.

Supplementary Material 1

## Data Availability

All the data and materials are available in this document.
