# Supplementary material for: Contextual effects: how to, and how not to, quantify them
Source: BMC Med Res Methodol. 2024 Feb 13;24:35. doi: 10.1186/s12874-024-02152-2 (PMC10863156; doi:10.1186/s12874-024-02152-2)
Supplement: Supplementary file 1 — Supplementary Material 1 [file 12874_2024_2152_MOESM1_ESM.docx]

## Supplement 1

**Proportional contextual effect (PCE)** [1, 2]

Theory from Hedges et al. [3] => response ratio

- $\bar{X_{i,j}}$: mean; i = 1 (treatment), i = 2 (placebo), j: can be f: follow-up or b: baseline

Overall treatment effect = $\frac{\bar{X_{1,f}} - \bar{X_{1,b}}}{{SD}_{change,pooled}}$

Contextual effect = $\frac{\bar{X_{2,f}} -\bar{X_{2,b}}}{{SD}_{change,pooled}}$

${SD}_{change,pooled}=\sqrt{\frac{\left( N_{1}-1 \right) * {SD}_{1,change}^{2} +\left( N_{2}-1 \right) * {SD}_{2,change}^{2}}{N_{1} + N_{2} - 2}}$ (standard deviation of the changes)

PCE = $(\frac{Improvement of the outcome in the placeo group}{Improvement of the outcome in active group})$ = $\frac{\frac{\bar{X_{2,b}} -\bar{X_{2,f}}}{{SD}_{change,pooled}}}{\frac{\bar{X_{1,b}} - \bar{X_{1,f}}}{{SD}_{change,pooled}}}$ = $\frac{d_{placebo}}{d_{active}}$

PCE is log-transformed for each study and SE are calculated according to Hedges et al. [3].

Pool log-transformed PCE log SE in Meta-analysis and back-transform via exponentiating.

## Supplement 2

**Logarithms in statistics** [4]

Log to base 10 of a number a is b where a = 10^b^ . This is written as b = log10a.

**Logs transform multiplication to addition and division to subtraction**

E.g. 100 * 1000 = 10^2^ * 10^3^ = 10^2^ + 10^3^ = 10^5^ = 100000 => log10(100 * 1000) = log10(100) + log10(1000) = 2 + 3 = 5.

Any form y=a *b * c *d can be transformed to log(y) = log(a) + log(b) + log(c) + log(d).

The same applies to division: y = a/b => log (a/b) = log(a) - log(b)

These can be back transformed by taking the antilog.

Logs can also be applied to other bases e. g. to e = 2.7183. These are written as ln(x).

The antilog of ln is to exponentiate with e. E. g. exp(ln(2) = 2.

Log-transformations are often performed to transform skewed distributions to more symmetrical distributions or transform curves to lines

# References

1. Zou K, Wong J, Abdullah N, Chen X, Smith T, Doherty M, et al. Examination of overall treatment effect and the proportion attributable to contextual effect in osteoarthritis: meta-analysis of randomised controlled trials. Ann Rheum Dis. 2016;75:1964–70.

2. Chen AT, Shrestha S, Collins JE, Sullivan JK, Losina E, Katz JN. Estimating contextual effect in nonpharmacological therapies for pain in knee osteoarthritis: a systematic analytic review. Osteoarthritis and Cartilage. 2020;28:1154–69.

3. Hedges LV, Gurevitch J, Curtis PS. THE META-ANALYSIS OF RESPONSE RATIOS IN EXPERIMENTAL ECOLOGY. 1999;80:7.

4. Bland JM, Altman DG. Statistics notes. Logarithms. BMJ. 1996;312:700.
